# Neural tracking of auditory statistical regularities in adults with and without dyslexia

**DOI:** 10.1093/cercor/bhaf042

**Published:** 2025-03-02

**Authors:** Hanna Ringer, Daniela Sammler, Tatsuya Daikoku

**Affiliations:** Next Generation Artificial Intelligence Research Center, Graduate School of Information Science and Technology, The University of Tokyo, 7-3-1 Hongo, Bunkyo-ku, Tokyo 113-8656, Japan; Research Group Neurocognition of Music and Language, Max Planck Institute for Empirical Aesthetics, Grüneburgweg 14, 60322 Frankfurt am Main, Germany; Research Group Neurocognition of Music and Language, Max Planck Institute for Empirical Aesthetics, Grüneburgweg 14, 60322 Frankfurt am Main, Germany; Department of Neuropsychology, Max Planck Institute for Human Cognitive and Brain Sciences, Stephanstraße 1a, 04103 Leipzig, Germany; Next Generation Artificial Intelligence Research Center, Graduate School of Information Science and Technology, The University of Tokyo, 7-3-1 Hongo, Bunkyo-ku, Tokyo 113-8656, Japan

**Keywords:** developmental dyslexia, electroencephalography, neural tracking, statistical learning

## Abstract

Listeners implicitly use statistical regularities to segment continuous sound input into meaningful units, eg transitional probabilities between syllables to segment a speech stream into separate words. Implicit learning of such statistical regularities in a novel stimulus stream is reflected in a synchronization of neural responses to the sequential stimulus structure. The present study aimed to test the hypothesis that neural tracking of the statistical stimulus structure is reduced in individuals with dyslexia who have weaker reading and spelling skills, and possibly also weaker statistical learning abilities in general, compared to healthy controls. To this end, adults with and without dyslexia were presented with continuous streams of (non-speech) tones, which were arranged into triplets, such that transitional probabilities between single tones were higher within triplets and lower between triplets. We found that the so-called Triplet Learning Index (ie the ratio of neural phase coherence at the triplet rate relative to the tone rate) was lower in adults with dyslexia compared to the control group. Moreover, a higher Triplet Learning Index was associated with better spelling skills. These results suggest that individuals with dyslexia have a rather broad deficit in processing structure in sound instead of a merely phonological deficit.

## Introduction

One of the key challenges of speech perception is to segment the continuous speech signal into meaningful units, such as words, syllables, or phonemes. Acquiring implicit knowledge that helps to accomplish this task plays a particularly crucial role during language learning, both during early childhood and when learning a second language later in life. Difficulties in acquiring and applying such knowledge to structure speech input, or acoustic input more generally, may be detrimental for the efficiency of speech perception, and therefore communication and well-being. In sequentially structured sound materials such as speech or music, short- and long-range statistical relationships that govern the order in which single elements (eg syllables or tones) are arranged within a longer sequence (eg words or melodic phrases) are a critical cue for the segmentation of acoustic input. In particular, segmentation occurs based on transitional probabilities between single elements, ie conditional probabilities for particular items to follow specific preceding items (Daikoku 2018). Sensitivity to the transitional probabilities between elements within a sound sequence is acquired through a powerful learning mechanism referred to as statistical learning (Saffran 2003). When presented with a constant stream of syllables that consisted of four trisyllabic artificial “words” repeated in a random order, 8-month-old infants rapidly learnt to segment the continuous syllable stream into distinct words based on transitional probabilities, which were high within words and low between words (Saffran et al. 1996). Automatic segmentation of stimulus streams based on transitional probabilities was subsequently also found for non-linguistic stimulus material in different sensory modalities, including sequences of pure tones (Saffran et al. 1999), visually presented shapes (Turk-Browne et al. 2005; Henin et al. 2021), and tactile vibration pulses at different stimulation sites (Conway and Christiansen 2005). Similar patterns of results were observed across different age groups from neonates (Teinonen et al. 2009; Bulf et al. 2011) to children and adults (Saffran et al. 1997), suggesting that statistical learning constitutes an innate capacity of the human brain. Moreover, it occurred largely implicitly, ie through passive exposure without explicit awareness that learning took place and without active access to what was learnt (Perruchet and Pacton 2006; Conway 2020), though some evidence suggests that learning of artificial words can be enhanced through explicit learning tasks, eg by providing linguistic information about the words such as written word forms (Chen et al. 2020).

At the neural level, perceptual segmentation of continuous acoustic streams based on their inherent (statistical) structure has been associated with neural tracking of the structure of the ongoing stimulation, ie the synchronization of oscillatory neural activity with the stimulation, sometimes referred to as neural entrainment (for a recent review on neural tracking as a measure of statistical learning in syllable streams, see Sjuls et al. 2023). Functionally, this mechanism was interpreted as the alignment of peaks in rhythmically fluctuating attention and perceptual sensitivity to the most relevant time windows within a continuous stimulus (Jones 1976; Large and Jones 1999; Henry and Herrmann 2014; Jones 2019). The frequency-tagging approach builds upon the tendency of the brain to automatically track periodically recurring events in continuous auditory stimulation and allows to experimentally test which perceptual units the brain responds to. Previous research using electroencephalography (EEG) combined with an adapted version of the statistical learning paradigm introduced above (Saffran et al. 1996) demonstrated that listeners’ brain activity synchronized with the rate of the trisyllabic words, which were separated by subliminal 25-ms pauses in structured syllable streams (Buiatti et al. 2009). More specifically, a peak in spectral power emerged at the word rate, whereas the peak at the syllable rate decreased while participants listened to the structured syllable streams (compared to random streams without a word structure; Buiatti et al. 2009). Strikingly, subsequent studies showed the same pattern of results even when trisyllabic words were not separated by pauses, ie when there were no acoustic cues in the syllable stream and segmentation must occur solely based on transitional probabilities between syllables (Batterink and Paller 2017; Batterink and Paller 2019; Choi et al. 2020). The so-called Word Learning Index (WLI), reflecting the strength of neural tracking (in terms of inter-trial coherence [ITC] of neural activity) at the word rate relative to the syllable rate, increased most strongly during the first few hundred word presentations. This suggests that sensitivity to the word structure in the syllable stream increased rapidly through passive exposure (Batterink and Choi 2021). Statistical learning of the word structure, as indexed by an increase of the WLI, was largely independent of listeners’ attentional focus (Batterink and Paller 2019) and occurred in both adults (Batterink and Paller 2017) and infants (Choi et al. 2020) alike. Concurrent tracking of syllables and words during statistical word learning was observed both in the presence and absence of additional prosodic cues, such as melodic contours (François et al. 2017) or stress patterns (Elmer et al. 2021) that corresponded to word boundaries. Other studies demonstrated robust neural tracking not only for syllables and words, but also for linguistic structures at longer time scales such as phrases or sentences (Ding et al. 2016), which was assumed to support parsing of the hierarchical syntactic structure of an utterance (Kazanina and Tavano 2023). Furthermore, a similar pattern of changes in neural tracking was observed for structured (compared to random) sequences of tones, suggesting that statistical learning shares similar neural signatures across different types of auditory stimulus material (Farthouat et al. 2017; Moser et al. 2021). Together, these results suggest that neural tracking does not merely reflect sensory processing of acoustic features, but automatic hierarchical integration of single elements within a sequence into meaningful perceptual units.

A growing body of research has suggested that statistical learning is impaired in individuals with developmental dyslexia, a specific learning disorder characterized by deficits in reading and spelling acquisition, despite normal intelligence and adequate school education (Lyon et al. 2003; Peterson and Pennington 2015). The worldwide prevalence of dyslexia is estimated at around 7% (Yang et al. 2022), and the condition is often associated with drastic lifelong consequences for professional success, social inclusion, and emotional well-being, including higher rates of anxiety, depression, and suicide (Huntington and Bender 1993; Tsovili 2004; Carroll and Iles 2006). While originally regarded as a primarily phonological deficit (Vellutino et al. 2004), evidence has accumulated that dyslexia is associated with a broader and fundamentally perceptual deficit that affects individuals’ abilities to process (statistical) regularities in sensory input and to use these regularities to enhance the efficiency of auditory perception. The idea of such a broader deficit is supported by the fact that impaired perceptual processing was not only shown for speech stimuli, but also for non-linguistic sound materials. For instance, compared to healthy controls, individuals with dyslexia showed less behavioral benefits from stimulus repetition in perceptual tasks such as tone frequency discrimination (Ahissar et al. 2006; Gertsovski and Ahissar 2022) and less neural adaptation to different types of repeated auditory stimuli such as voices, tones (Perrachione et al. 2016; Jaffe-Dax et al. 2017; Jaffe-Dax et al. 2018; Peter et al. 2019), and phoneme categories (Ozernov-Palchik et al. 2022). In particular, processing of more complex statistical regularities was found to be impaired in dyslexia, including the extraction of transitional probabilities in sequences of syllables or tones (Gabay et al. 2015) and learning of distributional information such as frequencies of occurrence of certain syllables (Kimel et al. 2022) or bimodal distributions along a continuum between two non-native phonemes (Vandermosten et al. 2019). Using the statistical learning paradigm introduced above, one study showed reduced neural tracking at the rate of artificial words within a structured syllable stream in individuals with dyslexia, as well as a positive correlation between the strength of neural tracking at the word rate and phonological awareness (Zhang et al. 2021). Another recent study investigated the neural correlates of auditory statistical learning more generally in adults with and without dyslexia by passively presenting them with continuous sequences of (non-speech) sounds, ie tones with different frequencies, that were arranged into triplets (Daikoku et al. 2023). Occasionally, the last tone of a triplet was either a statistical deviant, ie it had a low transitional probability given the first two tones of the triplet, or an acoustic deviant, ie it was presented from a different location. In healthy adults, both types of rare deviant events elicited a mismatch response in the event-related potential (ERP), suggesting that they had formed expectations about upcoming tones based on the recent stimulus history, which were violated by the incoming deviant tone. By contrast, in adults with dyslexia, the mismatch response to acoustic deviants was diminished and no mismatch response was elicited by statistical deviants, indicating that listeners failed to extract transitional probabilities between tones to predict upcoming tones (Daikoku et al. 2023).

Various theoretical frameworks offer different explanations for the link between impaired statistical learning and phonological deficits in dyslexia (Singh and Conway 2021), including insufficient perceptual anchoring to previous stimuli (Ahissar 2007), impaired serial order learning (Szmalec et al. 2011), inefficient temporal sampling of continuous stimulation (Goswami 2011; Goswami 2019), or a reduced auditory memory span (Banai and Ahissar 2018). While the exact neurocognitive mechanisms remain poorly understood, it is plausible to assume that weaker statistical learning skills in individuals with dyslexia reduce the efficiency of speech processing. On the one hand, impaired statistical learning impedes the anticipation of upcoming events based on statistical regularities within a continuous speech stream. On the other hand, inefficient segmentation of the continuous speech stream into meaningful units such as phonemes may hinder the build-up of robust representations of these units, which in turn impairs individuals’ abilities to recognize, categorize, and distinguish distinct phonemes.

The aim of the present study was to gain a better understanding of how adults with dyslexia process statistical regularities in acoustic (non-speech) sequences, in comparison to healthy adults. While the recent EEG study introduced above (Daikoku et al. 2023) focused on the automatic detection of rare deviant events that violate predictions based on transitional probabilities between tones within the continuous stream, the present study sought to investigate how auditory statistical regularities are neurally tracked during (passive) listening. To this end, we reanalyzed the EEG dataset by Daikoku et al. (2023), using the approach by Batterink and Paller (2017) to quantify sensitivity to the statistical triplet structure in the tone sequence based on ITC of oscillatory neural activity. We hypothesized that the quotient of ITC at the rate of triplets relative to ITC at the rate of single tones, ie an index corresponding to the WLI in the study by Batterink and Paller (2017), would be reduced in adults with dyslexia compared to the healthy control group. This would indicate that sensitivity to the statistical structure of auditory sequences is lower, and regularities are less closely neurally tracked in dyslexia. In particular, weaker neural tracking of non-speech sound sequences (instead of syllable sequences as used by a previous study; Zhang et al. 2021) would provide support for the idea of a broader perceptual rather than a merely phonological deficit. Moreover, we predicted that neural tracking of the statistical triplet structure would be associated with individual spelling and reading scores as assessed using standardized tests prior to the experiment, such that individuals who show stronger neural tracking also achieve higher spelling and reading scores.

## Materials and methods

This study is the reanalysis of an EEG dataset from a previous study (Daikoku et al. 2023). Below is the description of the procedures from the original experiment, followed by the explanation of the analysis methods of the present study.

### Participants

A total of 36 adult participants took part in the study.^1^ 17 of them (twelve female, five male; age *M* ± *SD*: 23.8 ± 4.2 years) had a formal diagnosis of developmental dyslexia, whereas the remaining 19 (13 female, six male; age *M* ± *SD*: 25.6 ± 3.3 years) formed the healthy control group. All participants had normal intelligence (IQ > 70; as assessed with a non-verbal intelligence test: Standard Progressive Matrices; Raven and Court 1998), no history of neurological or audiological disorders, and no diagnosis of general or specific language impairment. All of them were right-handed (as assessed with the Edinburgh Handedness Inventory; Oldfield 1971), native speakers of German, and had received a maximum of 5 years of formal musical training (besides music lessons at school). Approval of the experimental procedures was obtained from the local ethics committee of the Medical Faculty at Leipzig University (reference number: 2018/352) prior to the study. Participants provided written informed consent before the start of the study and received monetary compensation for their participation.

Before the actual EEG experiment, participants’ spelling and reading skills were assessed in a separate session to ensure that the groups indeed differed significantly in spelling and reading performance. As expected, members of the dyslexia group obtained lower scores than members of the control group in the German “Rechtschreibtest” (RST-ARR; Ibrahimović and Bulheller 2013), which required them to fill in missing irregular German words in a text that was read out by a native German person (*t*(34) = 9.71, *P* < 0.001, *d* = 3.373). Moreover, the dyslexia group showed reduced reading speed (*t*(34) = 5.29, *P* < 0.001, *d* = 1.766) and reading comprehension (*t*(34) = 4.91, *P* < 0.001, *d* = 1.637) compared to the control group, as assessed with the German “Lesegeschwindigkeits- und Verständnistest für die Klassen 5–12″ (LVGT 5–12+; Schneider et al. 2017). This test required them to read as much as possible of a continuous text within 4 min and to select from given words which of them fit some of the read text passages best.^2^

### Stimuli

The present study used the same sound stimuli as a previous study (Tsogli et al. 2019). A total of six Shepard tones based on different frequencies (F_3_ [174.61 Hz], G_3_ [196.00 Hz], A_3_ [220.00 Hz], B_3_ [246.94 Hz], C♯_4_ [277.18 Hz], and D♯_4_ [311.13 Hz]) were created by superpositioning nine sinusoidal components that were one octave apart. Each of the Shepard tones was combined with the sound of a percussion instrument (surdo, tambourine, agogô bells, hi-hat, castanets, and woodblock), with specific combinations of Shepard tones and percussion instruments counterbalanced across participants. For simplicity, these tones will be called tones A, B, C, D, E, F, and G in the remainder of the text. A separate set of six Shepard tones based on different frequencies was created for a short training phase prior to the start of the actual experiment. Additionally, one Shepard tone (based on C♯_5_ [554.37 Hz]) that was not combined with a percussive sound served as a target tone, which participants were asked to react to with a key press (see below). All tones had a duration of 220 ms, including a 10-ms rise and a 20-ms fall time, and a sampling frequency of 44,100 Hz.

**Fig. 1:**
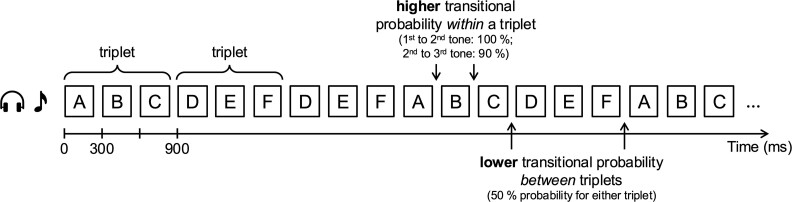
Schematic illustration of the structure of the tone sequence. Two triplets were presented in a random order, such that the probability for either triplet was 50% after any given triplet. By contrast, transitional probabilities within a triplet were higher (100% from the first to the second and 90% from the second to the third tone) than between triplets. Please note that this illustration of the tone sequence is simplified and only includes the standard triplets that were relevant for the present analysis; for a visualization of the complete paradigm including the different types of deviants, please refer to Fig. 1 in Daikoku et al. (2023).

The six tones (ie tones A, B, C, D, E, F) were arranged into two triplets (eg tones ABC and DEF), which were in turn combined to form a continuous tone sequence. Each of the six tones was presented an equal number of times, and assignment of specific tones to triplets as well as their position within triplets was counterbalanced across participants. Triplets were randomly ordered within the sequence, such that with two triplets the probability for either triplet was 50% after any given triplet, and unlike in most previous studies using similar statistical learning paradigms, the same triplet could occur more than once in a row. Each 220-ms tone was followed by a 80-ms silent inter-stimulus interval, ie both within and between triplets, resulting in a triplet duration of 900 ms and a constant tone rate of 3.33 Hz for the whole sequence. Thus, the transitional probabilities between individual tones were the only cue available to segment the continuous tone sequence (at the triplet rate of 1.11 Hz). Two types of deviants occurred occasionally within the tone sequence: The last tone of a triplet was either replaced by the last tone of the respective other triplet in 8% of the trials (statistical deviants), or was presented from a different speaker location in 18% of the trials (acoustic deviants). Both types of deviants occurred also combined as double deviants in 2% of all triplets. Since the aim of the present study was to investigate how listeners track statistical regularities in continuous auditory streams, deviants were not relevant to answer the research question of interest and only standard triplets without any deviant were included in the analysis. Figure 1 schematically illustrates the structure of the tone sequence, including only standard triplets that were relevant for the analysis reported here.^3^

### Procedure

Tone sequences were presented in six separate experimental blocks, each of which comprised 400 tone triplets and lasted ~6 min, resulting in a total of 36 min of exposure. During sound presentation, participants were seated inside an electromagnetically shielded chamber and EEG was recorded. Prior to the first experimental block, participants completed a short training phase (1 min) during which they could familiarize themselves with the task of pressing a key as fast as possible whenever they heard the target tone (ie the Shepard tone without a percussive sound). A total of 48 sounds were randomly replaced by the target tone throughout the experiment (eight per block), with target tone occurring equally often at each position within a triplet. To ensure that tracking of the (statistical) regularities of the tone sequence remained implicit, participants were not informed about the occurrence of deviants. In addition to the target detection task, they watched a silent documentary movie, which further diverted their attention away from the statistical regularities of the tone stream and reduced the probability that they focused on the triplet structure between the rare target occurrences.

### E‌EG data acquisition

Continuous EEG was recorded from 64 scalp electrodes mounted in an elastic cap in accordance with the extended international 10–20 system. In addition to the scalp electrodes, horizontal and vertical electrooculograms were recorded bipolarly from electrodes placed at the outer canthus of each eye and above and below the right eye. The electrode on the left mastoid served as an online reference. During preparation, electrode impedances were kept below 5 kΩ. Signals were amplified with a BrainAmp amplifier (Brain Products, Munich, Germany), online band-pass filtered between 0.25 and 1000 Hz, and digitized with a sampling rate of 500 Hz.

### Data analysis

EEG data were processed offline using the EEGLAB toolbox (version 2024.0; Delorme and Makeig 2004) in MATLAB (version R2024a; The MathWorks, Natick, MA). Subsequent statistical analyses were done in RStudio (version 4.2.2, RStudio Inc., USA).

### Pre-processing

Before pre-processing, noisy channels whose signal variance exceeded an absolute z-score of 3.0 were excluded and later spherically spline interpolated. Continuous data of the remaining channels were first high-pass and then low-pass filtered with Kaiser-windowed finite impulse response filters at 0.2 Hz (transition bandwidth: 0.4 Hz, maximum passband deviation: 0.001, filter order: 4638) and 30 Hz (transition bandwidth: 5 Hz, maximum passband deviation: 0.001, filter order: 372), respectively. To remove physiological and technical artifacts, we used an independent component analysis (ICA). ICA was computed on a copy of the data that was filtered with a 1-Hz high-pass filter (transition bandwidth: 0.5 Hz, maximum passband deviation: 0.001, filter order: 3710) to improve signal-to-noise ratio for decomposition (with the same 30-Hz low-pass filter as above), and down-sampled to 125 Hz to shorten computation time. We obtained ICA weights with an infomax algorithm implemented in EEGLAB’s runica function and transferred them to the dataset that was pre-processed with the final filter parameters. Artefactual components were identified and selected for removal using a combined automatic and manual procedure: Independent components were first classified using the IC Label plugin for EEGLAB (Pion-Tonachini et al. 2019), and components that were classified as eye blinks or movements, muscle activity, cardiac activity, line noise, or channel noise with a probability of at least 30% were automatically marked for rejection. Second, components classified as “other” were visually inspected with regard to their topography and spectral power distribution, and additionally selected for rejection if they were manually classified as one of the above-mentioned artifacts.

### ITC and triplet learning index

Neural tracking of statistical regularities, ie the triplet structure, in the continuous tone sequences was assessed using ITC of brain responses.^4^ As already mentioned above, triplets that contained any type of deviant (statistical, acoustic, or double) were excluded from the analysis. Furthermore, triplets that immediately followed a deviant triplet as well as triplets that contained a behaviorally relevant target tone were discarded. To generate epochs for ITC analysis, to-be-excluded triplets (ie triplets that contained a deviant, a target tone, or immediately followed a deviant triplet) were first removed from the continuous data. The remaining data (ie a sequence consisting only of standard triplets) were re-referenced to the algebraic mean of both mastoid electrodes. To attenuate baseline discontinuities where to-be-excluded triplets were removed from the continuous data, we then de-meaned the data at the level of single tones in a time window that ranged from 0 to 300 ms relative to each tone onset, ie covered the tone plus the silent inter-tone interval. Finally, for the computation of ITC, we generated non-overlapping epochs with a duration of 5.4 s, each of which covered six triplets (ie 18 individual tones). Epoching was done separately within each experimental block to make sure that no epochs were generated that contained block boundaries (corresponding to breaks within the experimental session). Any 5.4-s epoch with a peak-to-peak difference that exceeded 300 μV was discarded from the analysis, resulting in an average of 32 ± 1 (*M* ± *SD*) epochs per block for each participant. To improve signal-to-noise ratio, these data were averaged across nine electrodes within a fronto-central cluster (F1, F2, Fz, FC1, FC2, FCz, C1, C2, Cz), in line with the topographies shown by similar previous studies (eg Batterink and Paller 2017). Separately for each block, epochs were multiplied with a Hanning window to reduce 1/f noise, before Fast Fourier transforms (FFTs) were computed. In accordance with recent recommendations on how to best quantify neural tracking of sensory input (Benjamin et al. 2021), results of the FFT were averaged before ITC was estimated from the extracted phase angles. ITC coefficients were then extracted at the tone (3.33 Hz) and triplet (1.11 Hz) rate to compute the Triplet Learning Index (TLI; corresponding to the Word Learning Index reported by Batterink and Paller 2017), using the following formula:


$$ TLI=\frac{ITC_{triplet\ rate}}{ITC_{tone\ rate}} $$

The TLI is thought to reflect sensitivity to the triplet structure of the sequence, assuming that a shift towards relatively higher ITC at the triplet rate and relatively lower ITC at the tone rate over time indicates learning of the statistical regularity. We used a one-sided independent-samples *t*-test to statistically test the hypothesis that the dyslexia group shows a lower TLI (averaged across experimental blocks) compared to the control group, ie a weaker sensitivity to the statistical triplet structure.

To illustrate how the TLI changes as a function of statistical learning of the triplet structure over the course of the first block (during which most learning is expected to occur), we used a sliding time window approach (for a similar approach, see Batterink and Choi 2021). Specifically, we computed ITC in windows of 30 consecutive non-overlapping 5.4-s epochs (ie window length = 162 s), and shifted these windows in steps of 5.4 s. Time-resolved WLI was calculated and plotted as a function of the number of triplets presented so far. In line with previous literature (eg Choi et al. 2020; Batterink and Choi 2021), we fitted a logarithmic function (y = a * log(x) + b) to the TLI time course, separately for the dyslexia and the control group. The TLI time course was statistically compared between the control and the dyslexia group using a cluster-based permutation approach. First, independent-sample *t*-tests were computed for each time point, resulting in a time series of *t*-values. Second, we identified clusters of at least two adjacent data points at which the TLI differed significantly between the two groups (with *P* < 0.05), and computed the sum of *t*-values within each cluster. Finally, to determine the cluster threshold for statistical significance (at *P* < 0.05), a random distribution of cluster *t*-values was generated by permuting the assignment of individual time courses to the two groups and extracting the maximum cluster *t*-value from a total of 1,000 permutations.

To pinpoint the individual contributions of phase coherence at the tone and the triplet rate to the WLI changes over time, ITC as a function of the number of presented triplets was also plotted separately for the tone and the triplet rate (see Fig. S1). Further, we plotted the average TLI separately for each of the six experimental blocks to illustrate TLI changes throughout the whole experiment (see Fig. S2).

Moreover, we re-computed ITC for every single electrode (instead of the fronto-central cluster) to visualize the topographical distribution of ITC (on average across experimental blocks) at the triplet and syllable rate, respectively.

### Brain-behavior correlations

Finally, we computed brain-behavior correlations to assess the relationship between neural tracking of the statistical triplet structure in the tone sequences and spelling and reading performance (as assessed in the standardized tests explained above). To this end, raw test scores from the spelling test (RST-ARR) as well as raw scores of reading speed and reading comprehension from the reading test (LVGT 5–12+) were z-standardized. We then computed three separate Pearson correlations between each of the scores and the mean TLI across experimental blocks, respectively, and one-sided single-sample *t*-tests were used to test whether the correlation coefficients were significantly above zero.

## Results

On average, participants detected 94.3% (*SD*: 0.02%) of the targets in the target detection task, which suggests that they adequately attended to the task.

**Fig. 2:**
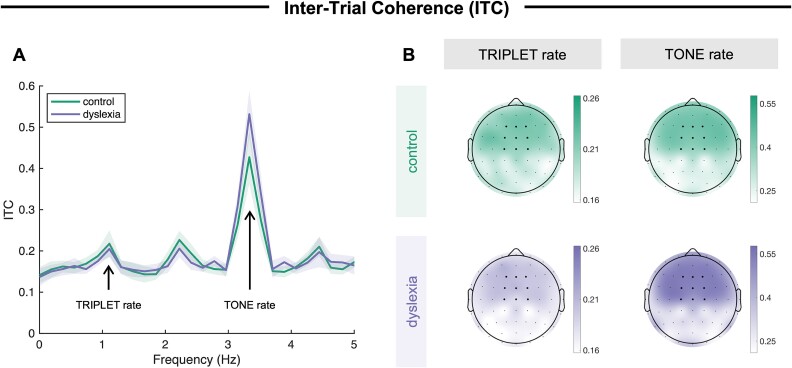
(A) Inter-trial coherence of neural activity (y-axis) across frequencies (x-axis) in the fronto-central electrode cluster of interest for the control and the dyslexia group. As marked in the plot, 3.33 Hz corresponds to the tone rate, and 1.11 Hz corresponds to the triplet rate. Shaded areas indicate ±1 standard error of means. (B) Topographies of ITC at the triplet rate (left panels) and the tone rate (right panels) for the control (upper panels) and the dyslexia (lower panels) group. Please note that different scales were used for the triplet and the tone rate. Black dots in the topography plots indicate electrode locations, and bold dots mark electrodes included in the fronto-central electrode cluster of interest.

As displayed in Fig. 2A, the largest peak in ITC in the fronto-central electrode cluster (across all experimental blocks) emerged at the tone rate (3.33 Hz) in both the control and the dyslexia group. In both groups, ITC was significantly increased at 3.33 Hz relative to control frequencies, ie mean ITC in symmetric frequency bins around the peak at 2.96 and 3.70 Hz (control: *t*(18) = 8.00, *P* < 0.001, *d* = 1.84; dyslexia: *t*(16) = 11.90, *P* < 0.001, *d* = 2.89). Clear peaks in ITC also occurred at the triplet rate (1.11 Hz), as well as at its harmonics (2.22 Hz and 4.44 Hz). These peaks were significant relative to symmetric control frequency bins around the peak at 0.75 and 1.48 Hz (control: *t*(18) = 2.98, *P* = 0.004, *d* = 0.68; dyslexia: *t*(16) = 4.40, *P* < 0.001, *d* = 1.07). ITC at the tone rate was lower for the control group compared to the dyslexia group (*t*(33) = 2.38, *P* = 0.023, *d* = 0.79), whereas ITC only tended to be slightly higher for the control group compared to the dyslexia group at the triplet rate (and its harmonics); however, the group difference was not significant at the triplet rate (*t*(33) = 0.66, *P* = 0.512, *d* = 0.22). The ITC effect at both tone rate and triplet rate was broadly fronto-centrally distributed across both groups, as shown in Fig. 2B, consistent with an origin in temporal (auditory) cortex. Thus, in both groups, participants’ brain activity became synchronized with the tone rate in the stimulation, and all participants picked up the statistical triplet structure in the tone sequence to some extent.

**Fig. 3:**
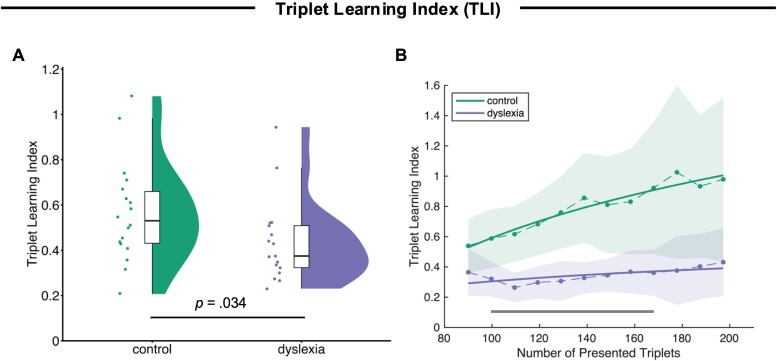
(A) Mean triplet learning index across experimental blocks for the control and the dyslexia group. Boxes indicate the inter-quartile range (around the median line), and single data points correspond to individual participants. (B) Triplet learning index (y-axis) as a function of the number of presented triplets (x-axis) throughout the first experimental block for the control and the dyslexia group. Dashed lines correspond to the actual data, and bold solid lines are logarithmic functions (y = a * log(x) + b) fitted to the data. Shaded areas indicate ±1 standard error of means (in the actual data). The gray horizontal bar indicates the time window in which the TLI differed significantly between the control and the dyslexia group, as tested using a cluster-based permutation approach.

Crucially, the TLI (displayed in Fig. 3) revealed that the relative weighting of synchronized activity at the tone rate and the triplet rate differed between the control and the dyslexia group: On average across experimental blocks, the TLI was significantly higher in the control group than in the dyslexia group (*t*(33) = 1.88, *P* = 0.034, *d* = 0.64)^5^, suggesting a relatively higher sensitivity to the triplet structure of the sequence (see Fig. 3A). As shown in Fig. 3B, this difference between groups emerged early throughout the first block, when TLI increased more steeply in control participants than in individuals with dyslexia as the number of presented triplets increased. A cluster-based permutation test revealed a significant cluster of differences in the time course of the TLI between the control and the dyslexia group (*z_cluster_* = 24.73, *p_cluster_* = 0.007), suggesting that the TLI over time in fact differed between individuals with and without dyslexia already throughout the first few minutes of exposure.

**Fig. 4:**
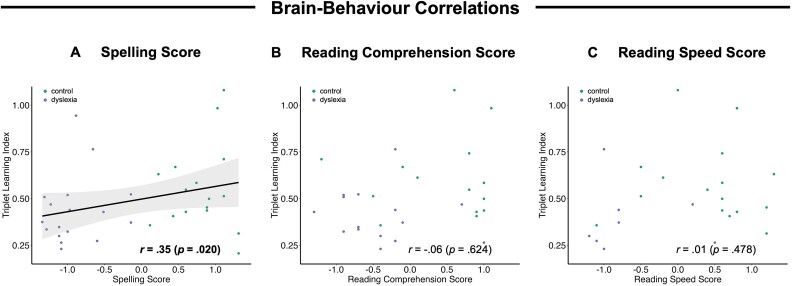
Correlation of the triplet learning index with individual spelling scores (A), reading comprehension scores (B), and reading speed scores (C), respectively. Shaded areas around the trend line for the significant correlation between the triplet learning index and the spelling score indicate the 95% confidence interval.

Finally, we analyzed the relationship between participants’ TLI across experimental blocks and their standardized scores in the spelling and reading test (Fig. 4). We found a significant positive correlation between participants’ TLI and their score in the spelling test (*r* = 0.35, *P* = 0.020). This suggests that participants with a higher spelling performance also showed stronger neural tracking of the statistical triplet structure in the tone sequence. No significant relationship was found between participants’ TLI and their reading speed (*r* = 0.01, *P* = 0.478) and reading comprehension (*r* = −.06, *P* = 0.624). The absence of a significant correlation for reading scores suggests that the positive correlation between participants’ TLI and spelling scores is not merely driven by group selection.

## Discussion

The current study aimed to compare neural tracking of the statistical structure of a sequence of tones between adults with and without dyslexia. Two groups of participants were passively presented with a continuous stream of tones, which were arranged into triplets, such that the transitional probabilities between tones were high within a triplet and low between triplets. Sensitivity to the statistical triplet structure was quantified with the TLI, ie the ratio of ITC at the triplet rate divided by ITC at the tone rate. While both groups did show an ITC peak at the triplet rate, suggesting that they picked up the triplet structure to some extent, the TLI was substantially lower in the dyslexia than in the control group. This indicates that individuals with dyslexia were relatively less sensitive to the statistical structure of the tone sequence, and their brains tracked the triplet rhythm less closely relative to the syllable rate. The difference between groups emerged early throughout the first experimental block, when the TLI increased much more steeply for healthy adults than for adults with dyslexia, pointing towards rapid learning of statistical regularities in the control, but not in the dyslexia group. Furthermore, the TLI was significantly correlated with individual spelling scores, such that stronger neural tracking of the triplet structure was associated with better spelling skills, but not with reading scores. The significant correlation suggests that the ability to acquire statistical regularities from the auditory environment is linked with (at least some) literacy skills that are relevant for the clinical diagnosis of dyslexia.

Overall, these results are in line with a large body of previous research that demonstrated impaired processing of (statistical) regularities in individuals with dyslexia across age groups. Earlier studies reported smaller behavioral benefits of stimulus repetition (Ahissar et al. 2006), diminished neural adaptation to different types of linguistic and non-linguistic sound materials (Perrachione et al. 2016; Jaffe-Dax et al. 2017; Jaffe-Dax et al. 2018; Peter et al. 2019; Ozernov-Palchik et al. 2022), reduced learning of more complex statistical regularities, including transitional probabilities (Gabay et al. 2015; Daikoku et al. 2023) and distributional statistics (Vandermosten et al. 2019; Kimel et al. 2022), and diminished neural tracking of the word structure within a syllable stream (Zhang et al. 2021). The current study extends these findings by comparing online neural tracking of statistical regularities in sequences of non-speech sounds between individuals with and without dyslexia. Specifically, the present results are in line with the hypothesis that the failure to detect rare, unexpected (statistical) deviant events that was observed in adults with dyslexia (Daikoku et al. 2023) may stem from their difficulties in tracking the regular statistical structure of the sensory input.

Interestingly, the increase in TLI, which we interpret as an online neural signature of statistical learning, was driven by a change in ITC at the rate of single tones rather than at the rate of tone triplets. Intuitively one may have expected that learning of the triplet structure of the tone sequence would be associated a gradual increase of neural tracking at the triplet rate; in fact, we observed a decrease of neural tracking at the tone rate (see Fig. S1 in the supplementary material, which shows the time course of ITC throughout the first experimental block separately at the tone and the triplet rate), which then resulted in an increase of the TLI (Fig. 3B). Similarly, the group difference in TLI (Fig. 2A) was a result of a lower ITC at the tone rate in the control group compared to the dyslexia group, rather than a difference in ITC at the triplet rate. There are some previous studies that also observed effects of statistical learning on neural tracking not only (or at all) at the triplet (or word) rate, but instead (also) at the tone (or syllable) rate: For instance, ITC was found to become gradually lower at the syllable rate while participants listen to a statistically structured syllable stream relative to a random stream (Batterink and Paller 2017), and participants’ attentional focus affected neural tracking at the syllable rate rather that the word rate (Batterink and Paller 2019). We suggest that the gradual decrease in neural tracking at the tone rate could be interpreted as increasing suppression of less relevant information (ie single tones), while more relevant information (ie the triplet structure) gains relatively more weight, assuming that processing information in chunks is a more efficient use of inherently limited perceptual resources. It may be plausible to assume that this mechanism of tuning down less important information in favor of up-weighting more important information operates less efficiently in individuals with dyslexia. Related to the previous finding that effects of attention were mainly reflected in neural tracking at the syllable rate (Batterink and Paller 2019), an alternative interpretation would be that attention allocation between the silent documentary, the auditory target detection task, and the whole sound sequence differed between groups (eg in a way that individuals with dyslexia less efficiently directed their attention away from the continuous sound sequence). Furthermore, on a more methodological note, these results highlight the importance of analyzing the relative weighting of neural tracking at the level of statistical chunks compared to single elements, which seems to be more informative than the absolute ITC per se.

Our finding of weaker relative tracking of statistical regularities in individuals with dyslexia has several implications both for theories on the neuro-cognitive underpinnings of developmental dyslexia and for possible clinical applications. From a theoretical point of view, the present results argue against a specific phonological deficit as the main cause underlying dyslexia (Vellutino et al. 2004). Instead, our findings support the idea of a somewhat broader perceptual impairment in processing statistical structure in sound. In particular, the presence of an effect despite the use of non-linguistic stimulus material indicates that the statistical learning deficit in individuals with dyslexia is not limited to speech input, but affects auditory processing more generally across a wider range of acoustic stimuli. Moreover, the correlation between neural tracking of the statistical structure of the tone sequence and individual spelling scores suggests that these abilities are interconnected. This relationship may be explained via phonological representations, which seem to be atypical in individuals with dyslexia: There is evidence that the perception of phonemes is less consistently categorical in both adults (Vandermosten et al. 2010) and children with dyslexia (Vandermosten et al. 2011) than in healthy control participants when they are asked to behaviorally classify stimuli along a synthetic phoneme continuum. Impoverished representations of phoneme categories may be grounded in deficits in statistical learning, which has been argued to play a crucial role for the acquisition of novel phoneme categories. More specifically, listeners are thought to acquire knowledge about the distribution of acoustic features through exposure to natural language, eg they learn that certain features (eg voice onset time, which distinguishes voiced and voiceless stop consonants such as/d/and/t/) are not randomly distributed across a continuum, but cluster around certain values to characterize and distinguish different phonemes (Myers 2014). Deficits in learning such distributional statistics, as they were reported for individuals with dyslexia, may result in impoverished representations of phoneme categories (Banai and Ahissar 2018), which in turn weaken phonological awareness, ie an individual’s knowledge about the sound structure of speech, which is an essential skill for reading and spelling development (Snowling 2000; Ziegler and Goswami 2005).

We can only speculate about why statistical learning was only correlated with individual spelling scores, but not with reading scores in the present dataset; however, it may be plausible to assume that adults have developed compensatory strategies such as the use of semantic context information, which is typically available during reading, but does not help during spelling. In the previous analysis of ERPs to statistical deviants in the same dataset, no correlation was found between ERP amplitudes and reading or spelling skills (Daikoku et al. 2023). The presence of a significant correlation of neural tracking with spelling skills in the present study suggests that continuous neural tracking is a more sensitive and direct measure of statistical structure learning than ERP responses to rare, unexpected statistical deviants.

From a more clinical and applied point of view, our present results suggest that statistical learning paradigms may be useful as an additional diagnostic tool in individuals with dyslexia or a screening tool in individuals at risk of dyslexia. The statistical learning paradigm has yielded robust results in children (Saffran et al. 1997), infants (Saffran et al. 1996), and even neonates (Teinonen et al. 2009), and here we show that neural correlates of statistical learning of non-linguistic auditory stimuli are related to participants’ spelling skills. Thus, such paradigms may be well-suited as a screening tool because they are relatively simple and efficient, do not require active behavioral responses, and do not rely on linguistic stimulus material; therefore, they may also be used in pre-verbal children, people with language impairments, and across different languages.

Although the present study provides an important proof of concept with regard to the link between statistical learning and literacy skills and highlight the potential of neural tracking as marker of statistical learning in individuals with dyslexia, a few methodological limitations should be considered when interpreting the current results. Whereas the stimulation sequence in previous studies that used the original statistical learning paradigm consisted of four to six triplets (eg Saffran et al. 1996; Saffran et al. 1999), this study used only two triplets. The reduced number of triplets inevitably resulted in immediate repetitions of the same triplet (at least) twice in a row within the stimulation sequence, which likely made the triplet structure more salient than in earlier studies and reduced the need to rely on transitional probabilities. While it is unlikely that the effect of interest, ie the difference in neural tracking between individuals with and without dyslexia, was driven solely by this fact, further studies using more complex sequences with a higher number of triplets are needed to generalize our claims with regard to statistical sequence learning in dyslexia.

## Conclusion

In conclusion, the present study demonstrated that neural tracking of statistical regularities in auditory (non-speech) stimulus sequences is reduced in adults with dyslexia compared to healthy adults, and that the magnitude of neural tracking of the statistical stimulus structure is associated with individual spelling scores. By combining an online measure of neural tracking of stimulus regularities and a statistical learning paradigm with non-linguistic sound material in individuals with dyslexia, these results both provide empirical support for theories claiming a link between statistical learning abilities and literacy skills and point out the potential use of such paradigms as a screening tool for dyslexia. To further explore the link between perceptual processing of statistical structure in sound and phonological skills and to derive new intervention approaches to ameliorate phonological deficits in dyslexia, future research may include groups of participants who are expected to show particularly good statistical learning abilities through extensive training, such as experienced musicians (Pesnot Lerousseau and Schön 2021), and involve longitudinal designs with different age groups across development.

## Supplementary Material

supplementaryMaterial_final_bhaf042

## Data Availability

Data is available from the corresponding author upon reasonable request.

## Footnotes

1 Additional five participants were tested, but excluded because they did not fulfill the inclusion criteria explained below. For further details, please refer to the original publication of the dataset (Daikoku et al. 2023).

2 For further details on the spelling and reading scores, please refer to the original publication of the dataset (Daikoku et al. 2023).

3 For further details on the complete paradigm, including the different types of deviants, please refer to the original publication of the dataset (Daikoku et al. 2023).

4 As a complementary measure, we also computed spectral magnitude. Spectral magnitude showed a virtually identical pattern of peaks across frequencies as did ITC, as expected. Therefore, we chose to report the ITC results in detail, since such as phase-based measure is less likely to depend on amplitude changes of the signal than power-based measures (Benjamin et al. 2021).

5 One participant from the control group was excluded from the analysis because their TLI differed from the group mean by more than 4 SD. Thus, the reported statistical results refer to data from 18 control participants and 17 individuals with dyslexia.
