# Supplementary material for: Neural tracking of auditory statistical regularities in adults with and without dyslexia
Source: Cereb Cortex. 2025 Mar 2;35(2):bhaf042. doi: 10.1093/cercor/bhaf042 (PMC11879346; doi:10.1093/cercor/bhaf042)
Supplement: supplementaryMaterial_final_bhaf042 [file supplementarymaterial_final_bhaf042.pdf]

Neural tracking of auditory statistical regularities in adults with and without dyslexia

Supplementary Material

Hanna Ringer[1,2*], Daniela Sammler[2,3], Tatsuya Daikoku[1]

[1] Next Generation Artificial Intelligence Research Center, Graduate School of Information Science and Technology, The University of Tokyo, Tokyo, Japan

[2] Research Group Neurocognition of Music and Language, Max Planck Institute for Empirical Aesthetics, Frankfurt am Main, Germany

[3] Department of Neuropsychology, Max Planck Institute for Human Cognitive and Brain Sciences, Leipzig, Germany

[*] Corresponding author:

Dr. Hanna Ringer

The University of Tokyo

7-3-1 Hongo, Bunkyo-ku, Tokyo 113-8656, Japan

Phone: +81-3-5841-1656

Email: ringer@g.ecc.u-tokyo.ac.jp

Running title: Neural tracking of auditory statistical regularities

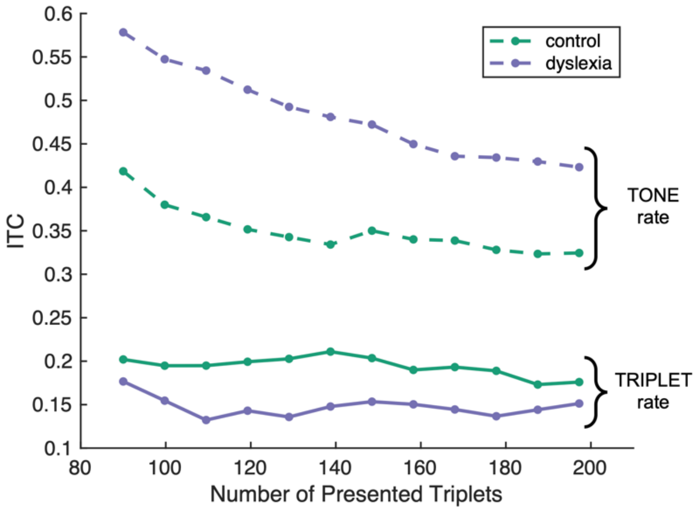

Figure S1. Inter-trial coherence (ITC; y-axis) at the tone (3.33 Hz; dashed lines) and the triplet (1.11 Hz; solid lines) rate as a function of the number of presented triplets (x-axis) throughout the first experimental block for the control and the dyslexia group.

In both groups, we tested whether ITC at the tone and at the triplet rate changed significantly throughout the first experimental block. More specifically, we computed the difference in ITC at the end relative to the beginning of the block (corresponding to the last minus the first data point of the time series in Figure S1) for each individual. These difference scores, with negative scores indicating a decrease and positive scores indicating an increase over time, were tested against zero using separate one-sample *t*-tests for the tone and the triplet rate in either participant group. In both the control ($t(18)$, = -2.65, p = .016) and the dyslexia group ($t(16)$, = -5.06, $p < .001$), ITC difference scores at the tone rate were significantly below zero, corresponding to a significant ITC decrease. Conversely, the change in ITC at the triplet rate was not significantly different from zero in neither the control ($t(18)$, = -0.74, p = .467) nor the dyslexia group ($t(16)$, = -1.07, p = .300). Together, these data suggest that the observed increase in TLI throughout the first block (as shown in Figure 3B) is accounted for by a change in ITC at the tone rate rather than at the triplet rate.

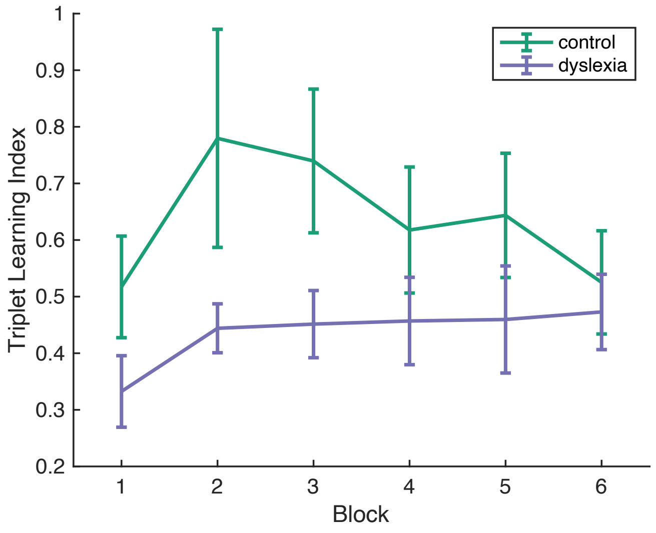

Figure S2. Triplet Learning Index (TLI; y-axis) in each of the six experimental blocks (x-axis) for the control and the dyslexia group. Error bars indicate ± 1 standard error of means. Across all blocks, TLI was higher in the control than in the dyslexia group. The strongest TLI increase happened from the first to the second block in both groups, and while there was not much change throughout the rest of the experiment in the dyslexia group, TLI tended to decrease in the control group, possibly related to adaptation throughout continued exposure.
